# Specific, or not specific, that is the question: Is Cre recombinase deleting your favorite gene only in cardiomyocytes?

**DOI:** 10.1016/j.jmccpl.2024.100082

**Published:** 2024-07-09

**Authors:** Onne A.H.O. Ronda, H. Llewelyn Roderick

**Affiliations:** Laboratory of Experimental Cardiology, Department of Cardiovascular Sciences, KU Leuven, Leuven, Belgium

## 1 Introduction

Tissue/cell-type specific knockout (KO) of a gene of interest (GOI) is a key methodology used in studies to establish its function. Nowhere is this more important than in the heart, where the development of tools to selectively modify the expression of target genes, specifically in cardiomyocytes (CM), has transformed our understanding of cardiac biology. Amongst the strategies employed, recombinase-based systems such as Cre-LoxP are commonly used to remove one or more exons to achieve genetic KO [1,2]. Different versions of these systems are implemented, which differ in the (specificity-determining) promoter and construct design [1,3]. The balance between sufficient recombinase transcription and tissue specificity has, however, not always been achieved leading to erroneous effects including cardiac pathology [3].

Cyclization recombinase (Cre) is a bacteriophage P1-originating enzyme that recognizes the ‘loxP’ sequence; an 8 bp spacer flanked by two unique 13 bp palindromic sequences [2,4]. A genomic region flanked by loxP positioned in the same orientation will result in Cre-mediated excision, whereas loxP in an opposing direction will result in Cre-mediated inversion [2]. Greater chromosomal distance between loxP sites decreases the recombination efficiency. Cre itself is constitutively active and nuclear localized. Cre (38 kDa), originally a prokaryotic protein, contains sequences resembling the bipartite nuclear localization signals (NLS) present in many eukaryotic nuclear proteins. To enhance nuclear targeting, some Cre fusion proteins have been designed with additional NLS (NLS-Cre). To achieve CM-specific expression, Cre has been positioned downstream of various promoters including *Myh6*, *Myh7*, *Tnnt2*, *Nkx2.5*, and *Mlc2*/*Xmlc2* although at >1100 publications, *Myh6*-driven Cre is the most widely used (Table 1). This promoter drives the expression of the thick filament protein α-MHC in both atrial and ventricular CM. In adult mice, contrary to adult humans, murine *Myh6* expression levels dominate over *Myh7*. *Myh6* is expressed in CMs from E8.5 allowing it to be used to induce genetic deletion after the initial stages of cardiac development are complete [2,5]. The *Myh7*- and the *Tnnt2* promoter, which are expressed in CM from E7.5, can also be used to generate CM-specific KOs. Owing to their expression in cardiac progenitors in the cardiac crescent from as early as E7.5, which continues into adulthood, Cre driven by the promoters of *Nkx2.5* and Xenopus *Mlc2* (*Xmlc2*) are useful tools for genetic manipulation during early cardiac development.

**Table 1:** Example Cre driver mouse strains useful in cardiology development and adult disease. MGI: Mouse Genome Informatics, TAM: tamoxifen, DOX: doxycycline. No. of ref., obtained from MGI (June 2024).

Driver	Synonym	Cre	Constitutive/inducible	Highest cited strain	MGI ID	No. of ref
Myh6	Alpha-MHC	WT Cre	*Constitutive*	B6.FVB-Tg(Myh6-cre)2182Mds/J	MGI:2386742	479
		CreERT2	TAM	Tg(Myh6-icre/ERT2)#Wet	MGI:5477906	18
		MerCreMer	TAM	B6.FVB(129)-A1cf^Tg(Myh6-cre/Esr1⁎)1Jmk^/J	MGI:3050453	481
Myh7	Beta-MHC	WT Cre	*Constitutive*	Tg(Myh7-cre)1Jmk	MGI:3054171	29
		CreERT2	TAM	Myh7^tm1(EGFP/cre/ERT2)Wtsi^	MGI:5633809	2
Tnnt2	cTnT	WT Cre	*Constitutive*	Tg(Tnnt2-cre)5Blh	MGI:2679081	87
		MerCreMer	TAM	Tnnt2^tm1.1(cre/Esr1⁎)Ccai^	MGI:5818274	4
		tetO-Cre	DOX	Tg(Tnnt2-rtTA,tetO-cre)1Wtp	MGI:3851371	15
Nkx2.5		WT Cre	*Constitutive*	Nkx2–5^tm1(cre)Rjs^	MGI:2654594	155
		CreERT2	TAM	Nkx2–5^tm1.1(cre/ERT2)Rard^	MGI:6791388	2
		tetO-Cre	DOX	Tg(Nkx2–5-tTA,tetO-EGFP/cre)1Smwu	MGI:5788432	2
XMLC2	Xenopus Myl2	WT Cre	*Constitutive*	Tg(myl7.L-cre)1118Tmhn	MGI:3712342	14
Myl2	MLC2v	WT Cre	*Constitutive*	Myl2^tm1(cre)Krc^	MGI:2385922	137

**Fig. 1:**
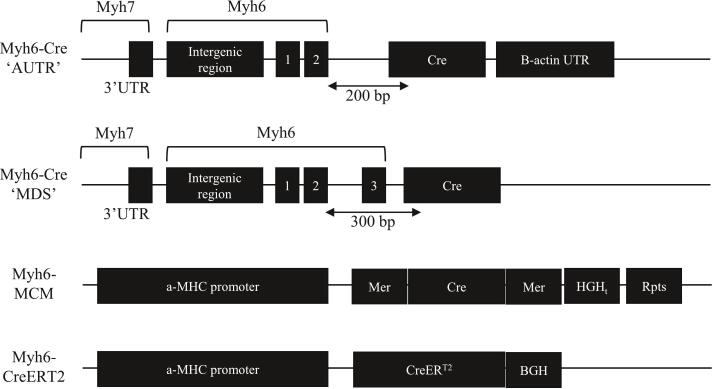
Cre construct layout, adapted from [1,4,8]. UTR: untranslated region, 1: exon 1, Cre: Cyclization recombinase, HGH_t_: human growth hormone transcriptional terminator, Rpts: duplicated sequences from the a-MHC promoter, Mer, and HGH_t_. BGH: polyadenylation signal from bovine growth hormone.

Cre-driven deletion of a GOI during development can give rise to adverse consequences ranging from lethality to functional compensation, thereby obscuring the *bona fide* activity of the GOI [3]. Such issues are circumvented through inducible versions of Cre [3,4], or viral-mediated (*e.g.* adeno associated virus; AAV) Cre delivery. Illustrating the success of this strategy, of the >4000 mouse strains harboring Cre, >1800 are described to be inducible. To endow inducibility upon Cre activity, it has been genetically fused with the ligand binding domains (LBD) of steroid hormone receptors such as the estrogen- (ER) and progesterone (PR) receptors. To avoid activation of these Cre constructs by endogenous steroids, their LBD are mutated to allow their activation only by exogenously applied synthetic ligands; tamoxifen (TAM; ER) and RU486 (PR) [4]. The Cre-ER fusion protein was created by fusing mutated (G521R) human ER LBD to P1 bacteriophage Cre: CreERT [6]. In the absence of TAM, CreERT interacts with the ubiquitous cytosolic Hsp90 complex. Addition of TAM, 4-hydroxytamoxifen, raloxifene, or ICI 182,780 (Fulvestrant) results in Hsp90 dissociation, revealing the CreERT NLS, allowing translocation to the nucleus [6,7]. To improve potency, additional mutations of the ER LBD have been introduced (G400V, M543A, and L544A) creating CreERT2, resulting in a 10× increased potency for TAM *versus* CreERT, allowing for lower TAM dosages and greater responsiveness [4]. Mutations of the murine ER LBD (G525R) similarly prevent estrogenic activation while maintaining TAM affinity: CreERTM (Cre/Esr1) [8]. To prevent ligand-independent recombinase activity, an ERTM has been appended to both the N and C termini of Cre creating the construct known MerCreMer (Fig. 1) [3,5,8]. Due to the dual presence of ERTM, MerCreMer should exhibit lower residual activity [3], making it more suited for long-term studies. Despite these modifications, ligand-independent activity (‘leakiness’) of the promoter may persist, and owing to the irreversibility and cumulative nature of DNA recombination is a major drawback of Cre-LoxP. Leakiness may also arise due to proteolysis of the Cre-ERT fusion protein, cryptic Cre mRNA splicing, the strength of the promoter driving Cre and Hsp90 transcription, as well as RNA/protein stability.

## 2 Testing tissue specificity

To test the temporal, and cell-type specificity of a Cre-driver, it is crossed with a ‘Cre reporter line’ such as *LacZ*, *Rosa26*^*Luc*^, or *Rosa26*^*tdTom*^. *B6.Cg-Tg(CAG-lacZ*)3*2And/J*, a LacZ reporter, permanently expresses LacZ following Cre-mediated excision of a 5′ STOP codon. LacZ translates into β-galactosidase; an enzyme capable of reacting with X-gal, creating blue insoluble product. FVB·129S6(B6)-*Gt(ROSA)26Sor*^*tm1(Luc)Kael*^/*J* and *B6.Cg-Gt(ROSA)26Sor*^*tm14(CAG-tdTomato)Hze*^*/J* (*Ai14*) are luciferase and tdTomato Cre reporters. These similarly begin permanently expressing luciferase or tdTomato after Cre-mediated removal of a 5′ STOP codon [9]. Luc and tdTom are observable on *ex*/*in-vivo* bioluminescence imaging and *ex vivo* fluorescent microscopy respectively. The CAG and Rosa26 promoters drive near-whole-body expression, making these reporters highly sensitive. Though, this also increases the occurrence of false positives. Tissue specificity is determined by both Cre and the LoxP, whereas Cre reporters only assess Cre tissue specificity. The emergence of high resolution single cell sequencing atlases covering murine development, which were not available at the time that most Cre lines were constructed, has provided new insights into expression patterns of these Cre lines, and in some cases identified their mis-expression in different tissues and cell types [1,9]. This valuable new information will guide not only the use of existing lines but also future Cre-line engineering.

## 3 Pitfalls and challenges

Ideally, Cre-LoxP itself should not affect the phenotype of interest, as this could lead to data misinterpretation [5]. For >2000 mouse strains catalogued in MGI, the constructs was randomly inserted into the genome, identifiable by the strain name “Tg()”. In the case of Myh6-MerCreMer, the insertion locus was identified, renaming this strain from B6.FVB(129)-Tg(Myh6-cre/Esr1*)1Jmk/J [8]. For *Myh6-MerCreMer*, the construct lies within the *A1cf* locus, meaning that these mice are heterozygous KO for *A1cf* when one *MerCreMer* copy is present [8]. Cre- and TAM toxicity are known and relevant issues, especially in cardiology [5,7]. High myocardial expression of Cre leads to dilated cardiomyopathy, likely due to recombination of pseudo-loxP loci, though may also pose cellular toxicity independent of recombination [10]. TAM can be cardiotoxic in certain Cre mice, although only in the presence of Cre expression [7]. TAM cardiotoxicity in *Myh6-MerCreMer* encompasses acute cardiomyopathy, which resolves after 21 days [7]. While a regimen involving up to 5 consecutive daily injections is routinely used herein to induce recombination, a single injection has also proved effective, with reduced deleterious consequences [7].

Davenport et al. [1] describe a unique case of non-specificity, illustrating the above points. They reported that 2 separate mouse lines, referred to as ‘AUTR’ and ‘MDS’ *Myh6-Cre*, the latter being the most-used constitutive *Myh6-Cre* line (MGI:2386742), also express Cre in the inner regions of some seminiferous tubules, where spermatids differentiate and mature [9]. Both lines use the same *Myh6* promoter, consisting of the *Myh7* 3′UTR, the *Myh7–6* intergenic region, and the first 2 exons of *Myh6*. However, ‘MDS’-*Myh6* also contains *Myh6*'s exon 3 (Fig. 1) [1]. They observed that male *Myh6-Cre*^+/−^;tdTom^Tg/−^, when crossed with WT females, produced whole-body fluorescent pups at a rate of ∼40 %, whereas only ∼1 % of pups were fluorescent when female *Myh6-Cre*^+/−^;tdTom^Tg/−^ were crossed with WT male [9]. Testicular *Myh6-Cre* is sufficiently expressed that Cre becomes active, leading to permanent whole body GOI^flox/−^ heterozygosity in F1 [9]. When heterozygous offspring are crossed, this leads to whole body GOI^−/−^ in a subset of F2 (GOI^flox/−^ × GOI^flox/−^ → 25 % GOI^−/−^), though this breeding strategy is generally not recommended for Cre-LoxP. They noted that ‘AUTR’ Yap^cKO^ was incompatible with life, whereas ‘MDS’ Yap^cKO^ maintained Mendelian inheritance patterns [1]. ‘AUTR’ expresses Cre in cardiomyocytes, and mosaically in brain, liver, pancreas, and testes [1]. A possible mechanism for this is that the ‘AUTR’ is associated with higher (ectopic) Cre protein abundance. This could be brought about *via* increased transcription through increased chromatin accessibility, an epigenetic landscape more conducive to transcription, presence of cis-regulatory elements or higher gene copy number. The mRNA levels may also be increased *via* greater RNA stability. Alternatively, the 3′ actin untranslated region of the ‘AUTR’ could enhance translation. The authors hypothesize that the gene-poor intergenic ‘MDS’ locus, *versus* AUTR's proximity to *GM40307*, could mean that ‘AUTR’ is influenced by cis-regulatory elements.

Some of the issues highlighted in the manuscript are also observed in another mouse line wherein Cre was integrated between the stop codon and the 3′ untranslated region of the endogenous *Myh6* gene. In this line, the tdTom reporter was detected in heart (both in CM and in smooth muscle cells), kidney, spleen, skeletal muscle, and lung [5]. This erroneous expression may result from the greater transcriptional activity of the endogenous *Myh6* locus than a *Myh6*-promoted gene integrated elsewhere in the genome which may not be optimal for transcription.

In conclusion, the above valuable new data from Davenport et al., and the examples provided herein illustrate the pitfalls and challenges associated with the Cre-LoxP system, particularly to genetically modify gene expression in CM. Particular issues to consider include: A) Whether the Cre is uniquely expressed in the desired cell type/tissue; B) Whether sensitive methodologies are available to detect Cre mis-expression; C) Whether cell-type specific inducible Cre lines exhibit leaky expression. D) Whether Cre expression is cardiotoxic. By considering these issues in experimental design through appropriate choice of Cre driver and mouse line, breeding strategy, and, if using an inducible Cre, TAM dosage (number of injections and dose), mechanistic insights can be obtained that will lead to discovery of new pathways and targets for therapy.

## Funding

OR is supported by a Postdoctoral Fellowship from the 10.13039/501100003130FWO (Fonds Wetenschappelijk Onderzoek, Research Foundation Flanders, 12D6523N). Work in the H.L.R. laboratory is supported by grants from the FWO (G063023N) and 10.13039/501100004040KU Leuven (C14/21/093; 20-VLIR-iBOF-027).

## CRediT authorship contribution statement

**Onne A.H.O. Ronda:** Writing – original draft. **H. Llewelyn Roderick:** Supervision, Writing – review & editing.

## Declaration of competing interest

The authors declare that they have no known competing financial interests or personal relationships that could have appeared to influence the work reported in this paper.
